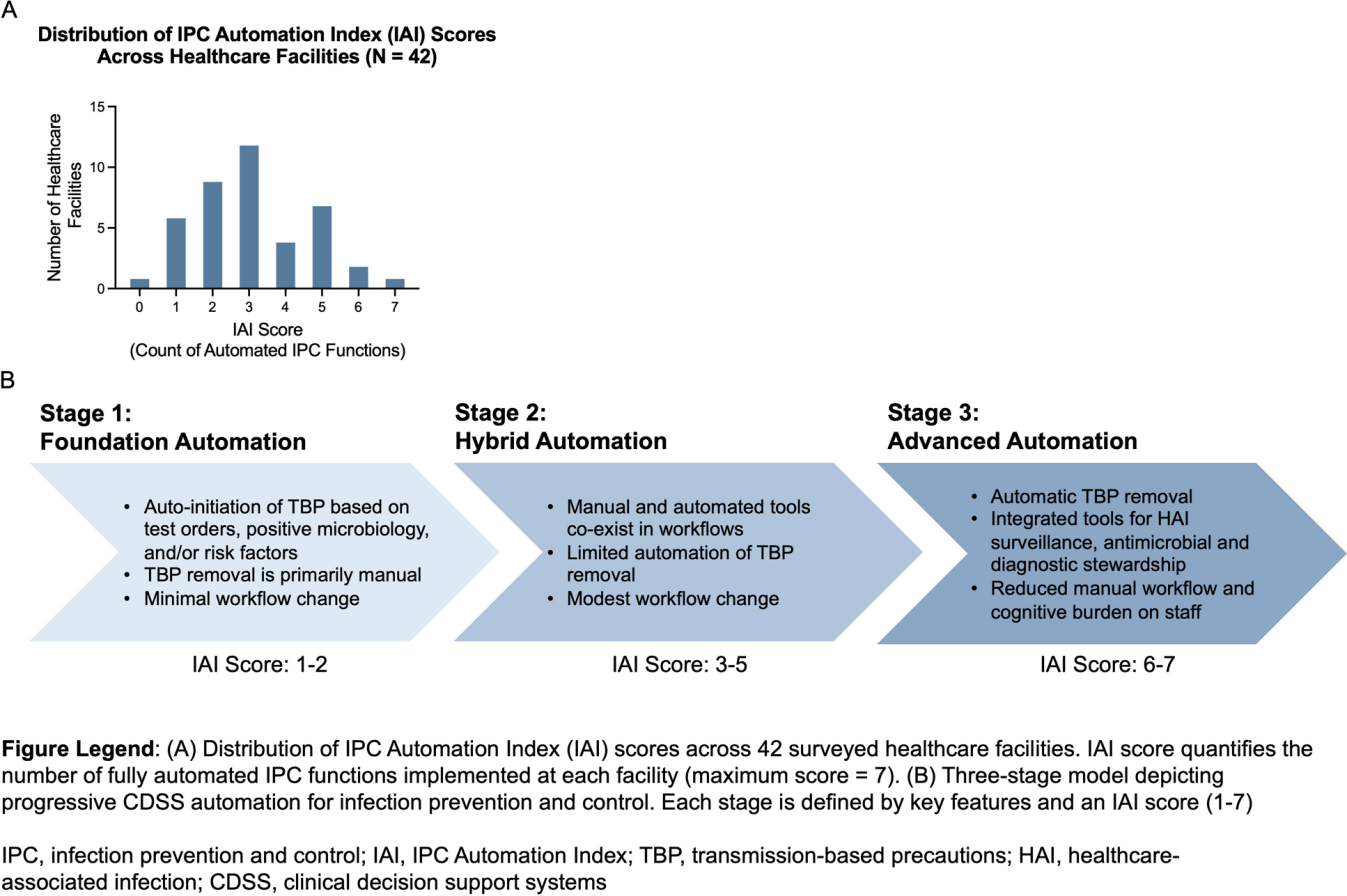# 272 Impact of Adding Anaerobic Coverage to Preoperative Antibiotics on Rates of Abdominal Hysterectomy (AH) Surgical Site Infections (SSI)

**DOI:** 10.1017/ash.2026.10449

**Published:** 2026-06-23

**Authors:** Chidi Akusobi, Jacob Lazarus, Caitlin M. Dugdale, Erica Shenoy

**Affiliations:** 1 Stanford University; 2 Massachusetts General Hospital; 3 Massachusetts General Hospital/Mass Eye and Ear; 4 Massachusetts General Hospital and Mass General Brigham

## Abstract

**Background:** Effective infection prevention and control (IPC) strategies are essential for reducing the risk of transmission of infection in healthcare settings. Traditionally, implementation and discontinuation of transmission-based precautions (TBP) have relied on manual review by infection preventionists (IPs) or physicians with IPC expertise. During the COVID-19 pandemic, rapidly evolving protocols and surges in patient volume strained IPC teams, highlighting the need for scalable, automated IPC workflows. Many healthcare systems have adopted clinical decision support systems (CDSS) built into the electronic health record to support non-IPC workflows; however, the extent of CDSS use for IPC is not known.? **Methods:** We conducted this national survey of healthcare facilities within the SHEA Research Network to broadly assess CDSS use in IPC, identify automated features, and define barriers to implementation. The survey captured IPC tasks supported by CDSS, including initiation and discontinuation of TBP and perceived impact of CDSS on clinical and operational outcomes. To enable cross-institutional comparisons, we defined an “IPC Automation Index” (IAI) score as the number of automated IPC functions at a facility across 7 core tasks assessed. **Results:** Fifty facilities completed the survey (42% response rate); of these, 42 (84%) reported using a CDSS tool for IPC. Although most used multiple CDSS tools for IPC, automation was largely concentrated at the front-end of workflows, particularly for automated initiation of TBP. In contrast, automated discontinuation of TBP was uncommon, with the majority of facilities relying on manual review. Over 55% of facilities had an IAI score of at least 2, indicating at least 2 fully automated IPC functions by CDSS (Figure A). Automation feature combinations varied widely, with 31 distinct configurations observed among 42 facilities and no dominant combination observed. Facilities with higher IAI scores were more likely to report perceived reductions in workload and cognitive burden among IPs. Finally, the most frequently reported barriers to CDSS adoption included personnel constraints (~45%), challenges integrating CDSS into the EHR (~43%), and financial limitations (~36%). **Conclusions:** This study provides the first national assessment of CDSS use for IPC, highlighting current practices, key barriers to adoption, and opportunities. We propose a CDSS automation model that conceptualizes IPC workflows along a continuum of low-to-high automation (Figure B). Anchored by the IAI score, this framework enables comparisons of CDSS use for IPC across healthcare facilities and highlights opportunities to advance CDSS adoption for IPC.

**Figure:**